# Enhanced Visible Light Photocatalytic Activity of Br-Doped Bismuth Oxide Formate Nanosheets

**DOI:** 10.3390/molecules201019189

**Published:** 2015-10-21

**Authors:** Xin Feng, Wen Cui, Junbo Zhong, Xiaoying Liu, Fan Dong, Yuxin Zhang

**Affiliations:** 1 Chongqing Key Laboratory of Catalysis and Functional Organic Molecules, College of Environment and Resources, Chongqing Technology and Business University, Chongqing 400067, China; E-Mails: fengxinsea@163.com (X.F.); 13527368524@163.com (W.C.); 2 Key Laboratory of Green Catalysis of Sichuan Institutes of High Education, Sichuan University of Science and Technology, Zigong 643000, China; E-Mail: liuxiaoying0202@foxmail.com; 3 National Key Laboratory of Fundamental Science of Micro/Nano-Devices and System Technology, College of Materials Science and Engineering, Chongqing University, Chongqing 400044, China; E-Mail: junbozhong@163.com

**Keywords:** Br-doping, BiOCOOH, NO removal, visible-light photocatalysis, charge separation

## Abstract

A facile method was developed to enhance the visible light photocatalytic activity of bismuth oxide formate (BiOCOOH) nanosheets via Br-doping. The as-prepared samples were characterized by X-ray diffraction, scanning electron microscopy, transmission electron microscopy, X-ray photoelectron spectroscopy, the Brunauer–Emmett–Teller surface area, UV-vis diffuse reflectance spectroscopy, photoluminescence spectra, and N_2_ adsorption-desorption isotherms measurement. The Br^−^ ions replaced the COOH^−^ ions in the layers of BiOCOOH, result in a decreased layer distance. The photocatalytic activity of the as-prepared materials was evaluated by removal of NO in qir at ppb level. The results showed that the Br-doped BiOCOOH nanosheets showed enhanced visible light photocatalytic activtiy with a NO removal of 37.8%. The enhanced activity can be ascribed to the increased visible light absorption and the promoted charge separation.

## 1. Introduction

Photocatalysis is a green technology with wide applications in solar energy conversion, environmental remediation and selective organic synthesis [1,2,3,4]. In the past few decades, TiO_2_, ZnO and other such kinds of photocatalytic materials have been investigated. However, they cannot utilize the solar energy effectively due to their wide band gap, which limits their visible-light induced practical applications [5,6,7,8,9]. To utilize the abundant visible light in solar light (about 48% fraction of sunlight) or indoor light, intensive investigations have been carried out for the development of visible-light photocatalysts.

Recently, bismuth-based materials such as Bi_2_WO_6_, Bi_2_MoO_6_, BiVO_4_, BiOX (X = Cl, Br, I), BiFeO_3_, Bi_2_Ti_2_O_7_, Bi_2_Sn_2_O_7_, (BiO)_2_CO_3_ and BiOCOOH *etc.* have been synthsized for photocatalytic applications [7,10,11,12,13,14,15,16,17,18,19,20,21,22,23,24]. Among these materials, BiOX (X = Cl, Br, I), (BiO)_2_CO_3_ and BiOCOOH have similar structures, with the general formula [Bi_2_O_2_][X*_m_*] (X = halogen or other groups) that usually possesses alternating [Bi_2_O_2_]^2+^ sheets and X slabs (*m* = 1, 2 or 3 rarely) [23]. Unfortunately, only BiOI and BiOBr are visible light responsive semiconductor photocatalysts, whereas BiOCl, (BiO)_2_CO_3_ and BiOCOOH can only be excited by UV light due to their wide band gap. Coupling a wide band gap semiconductor with a narrow band gap semiconductor to form heterostructures with a staggered alignment of band edges could greatly extend light responsive range and significantly improve the seperation of the photogenerated electrons and holes, thus effectively enhancing the photocatalytic activity and stability of single-component material. Heterostructured BiOCl/Bi_2_S_3_, BiVO_4_/Bi_2_S_3_, BiOI/Bi_2_S_3_, (BiO)_2_CO_3_/BiOI, BiOI/BIOCl, Bi_2_MoO_6_/(BiO)_2_CO_3_ and so on have been recently reported [25,26,27,28,29,30]. On the other, nonmetal doping has been widely employed to modify the band structure of wide band gap semiconductor. For example, N-doped, C-doped (BiO)_2_CO_3_ has been synthesized and showed enhanced visible light activity [19,20,21]. To the best of knowledge, little is known about the effect of Br-doping on BiOCOOH.

In previous reports, Chen’s group prepared BiOCOOH with different shape through a facile and template-free solvothermal process in different solvents, including spherical-like, sponge-like, tremella-like and flower-like hierarchical nanostructures [22]. Also, a possible formation mechanism involved with Ostwald ripening and self-assembly process was proposed. The sponge-like BiOCOOH hierarchical nanostructures exhibited the highest photocatalytic activity among the as-synthesized BiOCOOH hierarchical nanostructures [22]. Afterwards, Zhu’s group synthesized ultralong BiOCOOH nanowires via a simple solvothermal route, which exhibited highly efficient adsorption for anions including Cr(VI) and MO due to its positively charged surface, large porosity, and good dispersion in water [24]. To improve the photocatalytic activities of pure BiOCOOH, Wang’ group fabricated the BiOI/BiOCOOOH composites by a facile partial ion exchange method between BiOCOOH and KI with ultrasonic reaction at acidic condition [23].

In this work, to extend the light absorption spectra of BiOCOOH into visible light region, we developed a simple ion-exchanging method for Br-doped BiOCOOH. The photocatalytic activity of the samples is evaluated for removal of NO at ppb-level under visible light. The result indicated that the Br-doped BiOCOOH exhibited more efficient visible light activity than pure BiOCOOH as Br-doping could improve the visible light absorption and charge separation. This work has demonstrated a new strategy for modification of layered photocatalyst via ion exchange.

## 2. Results and Discussion

### 2.1. Phase Structure

**Figure 1:**
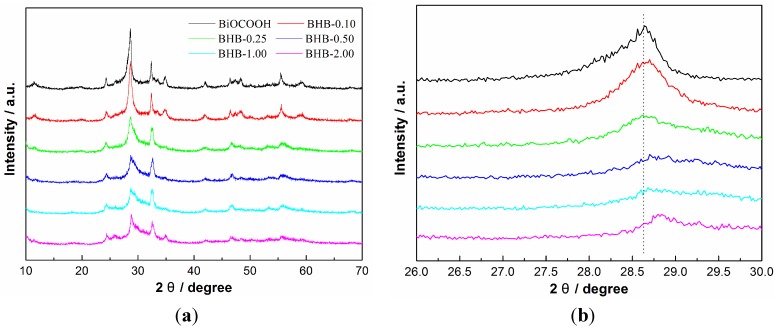
XRD patterns of as-prepared BiOCOOH and BHB with different Br contents (**a**) and the enlarge view (**b**).

XRD was used to characterize the phase structure of the as-prepared samples. Figure 1a shows the XRD patterns of BiOCOOH and BHB with different Br content. All the samples are well crystallized. The diffraction peaks of the samples can be indexed to the BiOCOOH (JCPDS-ICDD Card No. 35-0939). The peak at around 2θ = 28.6° corresponds to (102) plane of BiOCOOH, and this peak intensity becomes lower as the molar ratio of Br contents is increased from 0 to 2.00. With the increasing molar ratio of Br, this peak has a slight shift to a higher angle (Figure 1b), indicating a decreased layer distance. The decreased peak intensity and the shifting of the peak position can be ascribed to the doping of Br^−^. The Br^−^ ions may replace the COOH^−^ ions in the layers of BiOCOOH and co-ordinate with oxygen atoms of [BiO]^+^ layer. Note that no BiOBr phase can be detected, indicating that the Br is doped into the crystal structure of BiOCOOH. As the size of Br^−^ ions is smaller than that of COOH^−^ ions, the partial replacement of COOH^−^ ions with Br^−^ ions would result in a decreased layer distance.

### 2.2. Morphological Structure

**Figure 2:**
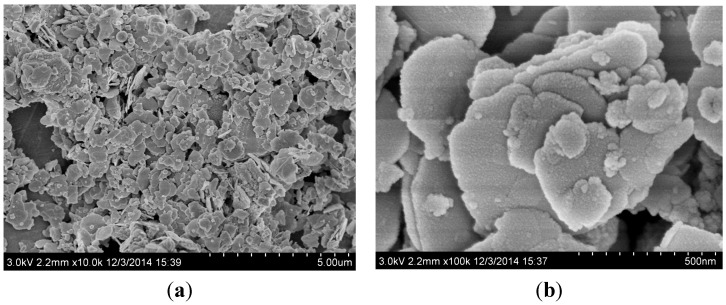
The SEM images of BiOCOOH.

**Figure 3:**
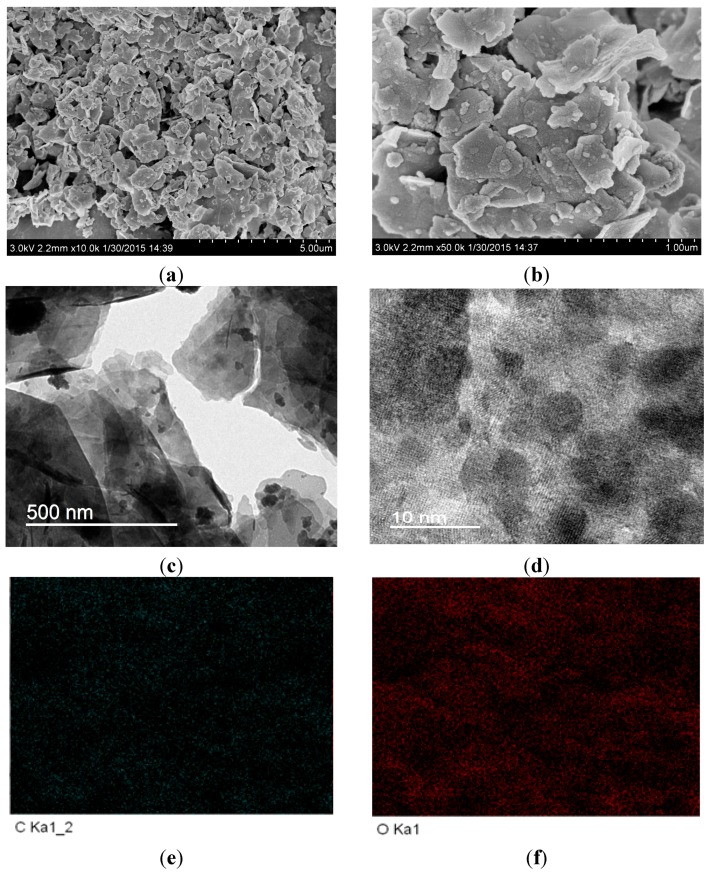

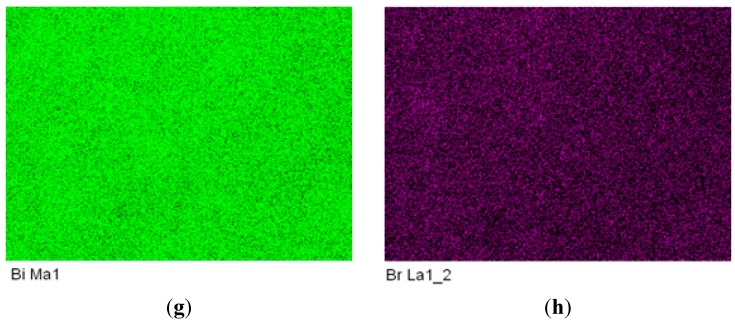
The SEM (**a**,**b**); TEM (**c**) and HRTEM (**d**) images and EDS mapping (**e**–**h**) of BHB-0.25.

The morphology and microstructure of the obtained samples were characterized by SEM, TEM and EDS mapping. In Figure 2a,b, we can observe that the pure BiOCOOH sample consists of many two-dimensional (2D) nanosheets. These nanosheets are in different sizes. Compared with the pure BiOCOOH nanosheets, the morphology of Br-doped BiOCOOH (BHB-0.25) is similar (Figure 3a,b), which implies that Br-doping has little influence on the morphology of BiOCOOH nanosheets. The lattice spacing of 0.310 nm can be well assigned to the (012) plane of BiOCOOH (Figure 3c,d). The EDS mapping (Figure 3e–h) indicates the elemental distribution of C, O, Bi and Br. The doped Br is uniformly distributed across the sample.

### 2.3. Local Chemical Structure by XPS

**Figure 4:**
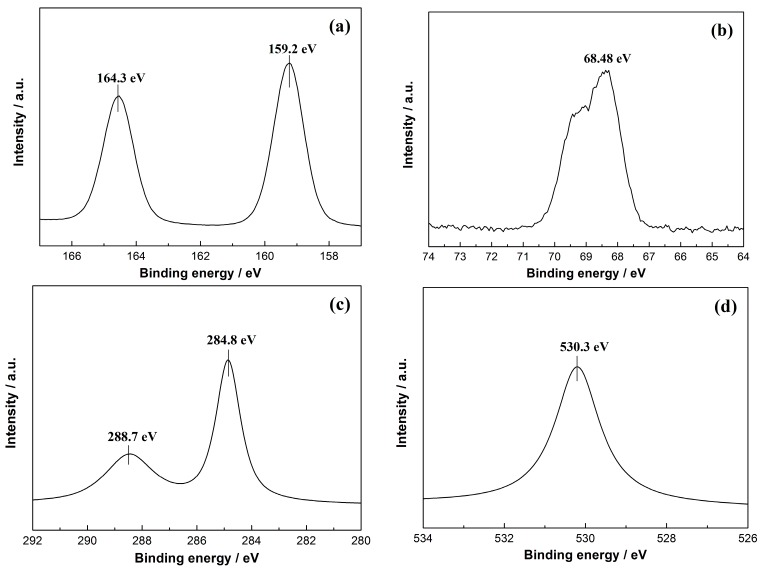
The XPS spectra of BHB-0.25, Bi4f (**a**); Br (**b**); C1s (**c**) and O1s (**d**).

XPS measurement was performed to determine the chemical composition of BHB-0.25. The peaks (Figure 4a) with binding energies at 159.2 and 164.3 eV can be ascribed to Bi 4f_7/2_ and Bi 4f_5/2_ [20,21,22,23]. The Br 3d XPS spectra with a peak at 68.48 eV (Figure 4b) can be ascribed to the doped Br [31,32]. The atomic molar ratio of Br in BHB-0.25 is 5.90%. As the BiOCOOH has a layered crystal structure, the Br^−^ ions could enter into the space between the layers, and then replace the COOH^−^ ions and co-ordinate with oxygen atoms. However, this issue needs to be investigated theoretically in the future work. Figure 4c shows that the C 1s spectra which can be fitted with two peaks. The peaks located at 284.8 eV are usually assigned to adventitious carbon, and another weak peak at 288.7 eV corresponds to carboxyl carbon. The O1s XPS spectra at 530.3 eV (Figure 4d) can be ascribed to the Bi–O bond in [BiO]^+^ slabs.

### 2.4. BET Surface Areas and Pore Structure

**Figure 5:**
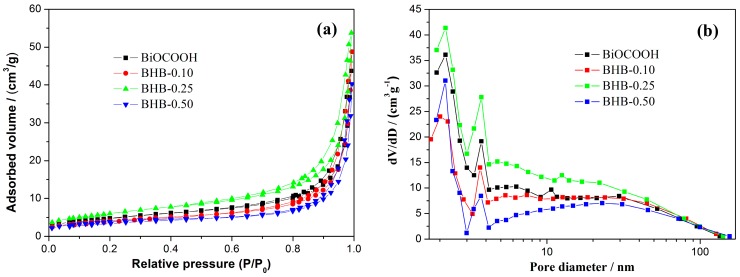
N_2_ adsorption-desorption isotherms (**a**) and pore size-distribution curves (**b**) of BiOCOOH and BHB-X.

Figure 5 shows the nitrogen adsorption-desorption isotherms and the corresponding pore size distribution curves of the samples. According to the Brunauer-Deming-Deming-Teller (BDDT) classification, the majority of physisorption isotherms can be classified into six types. Typically, the BiOCOOH and BHB-X both have an isotherm of type IV. The BHB-0.25 displays a higher absorption at the relatively high pressure comparing with others, suggesting the presence of mesopores in this sample. As no mesopores are contained in the BiOCOOH based nanosheets. These mesopores should be the void space between the nanosheets. This result is also demonstrated by the pore-size distribution curves. Figure 5b shows that the four samples have the peak pore sizes at 1.2 and 2.8 nm. The S_BET_, pore volume and peak pore diameter are summarized in Table 1. The sample with high surface area would facilitate the exposure of active sites and promote the photocatalysis efficiency.

**Table 1:** The *S*_BET_, pore volume, peak pore diameter, NO removal ratio of the samples.

Sample	*S*_BET_ (m^2^/g)	Pore Volume (cm^3^/g)	Peak Pore Diameter (nm)	NO η (%)
BHB	12.98	0.067	1.2/2.8	0
BHB-0.10	14.69	0.075	1.2/2.8	31.4
BHB-0.25	21.93	0.083	1.2/2.8	37.6
BHB-0.50	13.64	0.062	1.2/2.8	33.5

### 2.5. Light Absorption and Charge Separation

**Figure 6:**
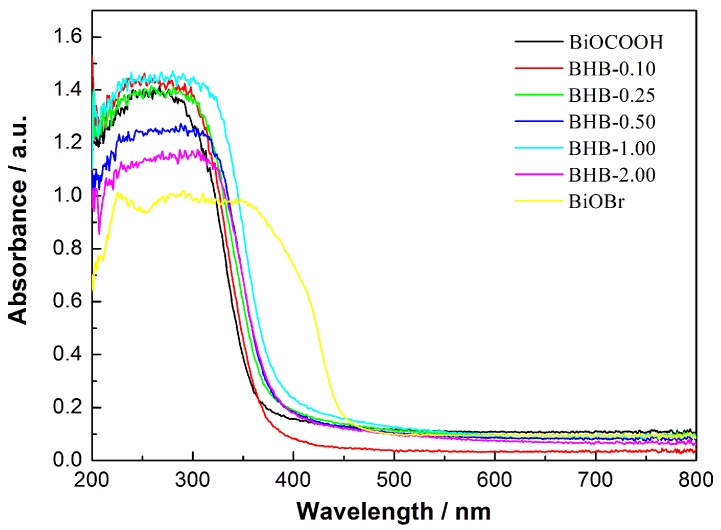
UV-vis DRS spectra of BiOCOOH, BiOBr and BHB with different Br contents.

The pure BiOCOOH has a white color, while the samples after Br-doping are yellow. With the increased molar ratio of Br-doping, the color is changed from pale yellow to dark yellow. From the UV-vis DRS spectra (Figure 6), we can observe that the pure BiOCOOH displays the absorption edge around 375 nm.

**Figure 7:**
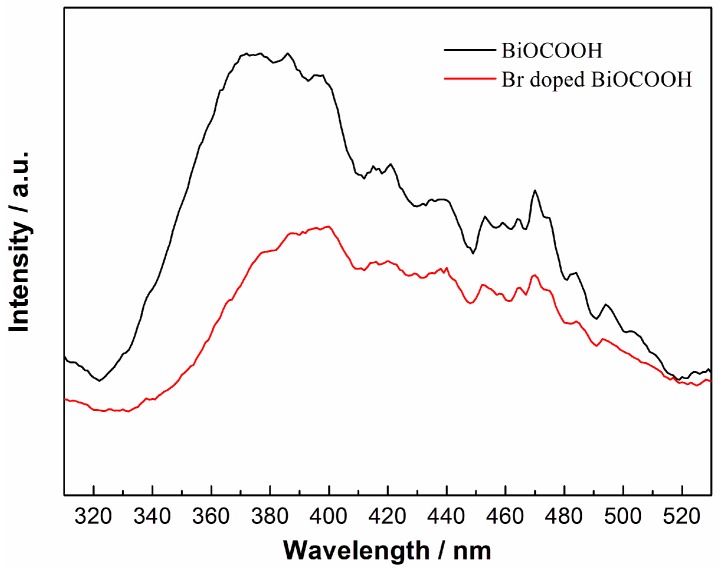
PL spectra of BiOCOOH and Br doped BiOCOOH (BHB-0.25).

With the increasing molar ratio of doping from 0.10 to 0.50, the absorption edge is red-shifted to around 400 nm and the light absorption spectra are broadened to 600 nm in visible light region. The enhanced visible light absorption should be ascribed to Br-doping which leads to narrowed band gap. As is known for nonmetal-doped TiO_2_, non-mental doping could narrow the band gap of TiO_2_. The result presented here may be similar to nonmetal-doped TiO_2_. To measure the recombination rate of the photogenerated electron-hole pairs, PL spectra is applied. Usually, a low PL intensity indicates a high charge separation efficiency [33]. As can be seen in Figure 7, pure BiOCOOH gives a high peak intensity with the excitation wavelength of 280 nm. With Br-doping, the PL peak is significantly decreased, which indicates a largely depressed recombination of photoinduced electron-hole pairs. The result implies that the Br-doping can improve separation of photogenerated electron-hole pairs and thus enhance the photocatalytic performance.

**Figure 8:**
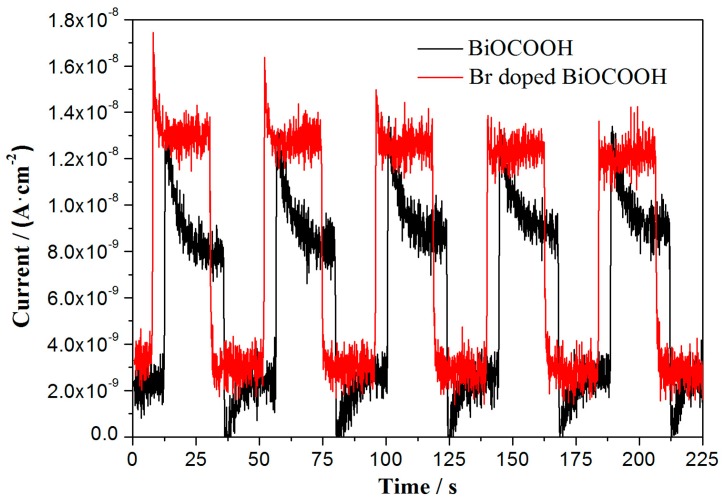
Photocurrent generation for BiOCOOH and Br-doped BiOCOOH (BHB-0.25) under visible light irradiation (λ > 420 nm, [Na_2_SO_4_] = 0.5 M).

Photocurrent generation was carried out for BiOCOOH and Br-doped BiOCOOH (BHB-0.25) electrodes to evaluate the charge separation efficiency. Figure 8 shows that the steady and prompt photocurrent generation is obtained during on and off cycles of visible light illumination.

The BiOCOOH sample shows certain photocurrent response. The charge transport proceeds quickly, making the changes of “on” and “off” currents are nearly vertical. It is significant to observe that the photocurrent of the Br-doped BiOCOOH electrode is higher than that of the pure BiOCOOH electrode. The photocurrent enhancement of Br-doped BiOCOOH can be ascribed to the enhanced photo-generated electrons/holes separation. This is also consistent with PL result.

### 2.6. Visible Light Photocatalytic Activity

**Figure 9:**
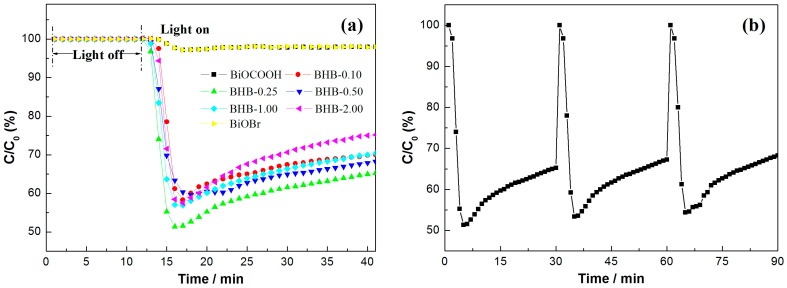
Visible light photocatalytic activity of pure BiOCOOH, BiOBr and BHB with different Br contents (**a**) and repeated visible light photocatalytic runs of BHB-0.25 (**b**) for NO removal in air.

NO with concentration at ppb level is one of the typical air pollutants, which is very stable and cannot be photolyzed under light irradiation without photocatalysts. Figure 9a shows the variation of NO concentration (C/C_0_%) with irradiation time over the as-prepared samples in dark and under visible light irradiation. As previous reported, BiOCOOH nanosheets exhibits little visible light activity due to the large band gap. The concentration of NO is stable before light-on. All the Br-doped samples exhibit decent visible light photocatalytic activity for NO removal when the light is turned on. When the *X* value is increased to 0.25, the NO removal ratio is increased to as high as 37.8%. Further increasing the *X* value to 2.00, the NO removal ration is decreased. Note that the NO removal ratio is gradually decreased due to the occupation of the surface with the final products. The photocatalytic oxidaiton raction of NO is shown in Equations (1)–(4) [33,34]. The final oxidation products (nitric acid or nitrate ions) can be simply washed away by water washing:

(1) $$\mathrm{NO}\;+\;2•\mathrm{OH}\;\to \;NO_{2}+\;H_{2}O$$

(2) $$NO_{2}\;+\;•\mathrm{OH}\;\to \;N{O_{3}}^{-}+\;H^{+}$$

(3) $$\mathrm{NO}\;+\;NO_{2}+\;H_{2}O\;\to \;2\mathrm{HN}O_{2}$$

(4) $$\mathrm{NO}\;+\;•{O_{2}}^{-}\to \;N{O_{3}}^{-}$$

As demonstrated in Figure 6, Figure 7 and Figure 8 Br-doping could increase the visible light absorption and enhance the charge separation of BiOCOOH, which in turn directly contribute to the promoted visible light photocatalytic activity of Br-doped BiOCOOH.

To put the as-prepared samples into practical applications, the photocatalytic stability of the samples should be examined. An ideal photocatalyst should maintain photochemical stability and durability under repeated irradiation so that it can be reused [35,36]. Figure 9b shows the repeated photocatalytic activity of BHB-0.25 under visible light irradiation. The BHB-0.25 does not show obvious deactivation after three recycles.

## 3. Experimental Section

### 3.1. Synthesis of BiOCOOH Nanosheets

All chemicals used in this study were analytical grade. In a typical fabrication, to bismuth nitrate pentahydrate (0.48 g) was added dimethyl formanide (DMF, 30 mL), sonicated for 30 min at room temperature and then centrifuged to get rid of undissolved precipitates. Then the resulted solution was transferred into a 50 mL autoclave Teflon and hydrothermally treated at 120 °C for 24 h. The obtained solid sample was filtered, washed with water and ethanol for three times, and dried at 60 °C for 12 h to get final BiOCOOH.

### 3.2. Synthesis of Br-Doped BiOCOOH Nanosheets

In a typical process, BiOCOOH (0.81 g) was added into distilled water (60 mL), and then ultrasound dispersed for 30 min. Then KBr aqueous solution (40 mL) with concentraiton of 0.0075, 0.01875, 0.0375, 0.0750, and 0.1500 g/mol was added to the above solution under continuous stirring for another 2 h, respectively. The sample obtained was filtered, washed four times with water and ethanol and dried at 60 °C for 12 h to afford the final products. The molar ratio of KBr to BiOCOOH is 0.10, 0.25, 0.50, 1.00 and 2.00, respectively. Accordingly, the resulted products were labeled as BHB-0.10, BHB-0.25, BHB-0.50, BHB-1.00 and BHB-2.00, respectively.

### 3.3. Synthesis of BiOBr

Deionized water (85 mL), glacial acetic acid (45 mL), and bismuth nitrate (14.55 g, 30 mmol) were placed into a 250 mL beaker and stirred at room temperature for 15 min until a clear, transparent solution was formed. Then cetyltrimethylammonium bromide (CTAB, 10.93 g dissolved in 40 mL of water, 30 mmol) was added to the above solution in one batch, and the mixture was stirred for an additional 30 min at room temperature. The precipitate thus formed was filtered and washed several times with ethanol and water to remove the non-reactive species. The solid was then dried for further use.

### 3.4. Characterization

The crystal structure of the as-obtained sample were analyzed by X-ray diffraction with Cu-Karadiation (XRD: model D/max RA, Rigaku Co., Tokyo, Japan). The morphological structure were examined by transmission electron microscopy (TEM: JEM-2010, JEOL, Tokyo, Japan). X-ray photoelectron spectroscopy with Al Kα X-rays (hν = 1486.6 eV) radiation operated at 150W (XPS: Thermo ESCALAB 250, Fisher Scientific Inc., Waltham, MA, USA) was used to investigate the surface properties. The shift of the binding energy due to relative surface charging was corrected using the C1s level at 284.8 eV as an internal standard. The Brunauer–Emmett–Teller (BET) surface area and the pore size distribution of the products were identified by an ASAP 2020 apparatus (Micromeritics, Atlanta, GA, USA). All the samples were degassed at 150 °C prior to measurements. The UV-vis diffuse reflection spectra were obtained for the dry-pressed disk samples using a Scan UV-vis spectrophotometer (UV-vis DRS: UV-2450, Shimadzu, Tokyo, Japan) equipped with an integrating sphere assembly using 100% BaSO4 as reflectance sample. The photoluminescence (PL) spectra were measured with a fluorescence spectrophotometer (PL: FS-2500, Toshiba, Tokyo, Japan) using a Xe lamp as an excitation source with optical filters. The photocurrent was measured using an electrochemical system (CHI-660B, Chinehwa, Shanghai, China), using the FTO glass with the as-prepared samples coated on the working electrode, saturated calomel electrode as the reference electrode, and Pt wire as the counter electrode. For the photocurrent measurement, the working electrode was irradiated by a 300 W Xe lamp with a 420 nm cut-off filter. The photocurrent–time dependence at open circuit potential was measured in 0.5 M Na_2_SO_4_ under chopped illumination.

### 3.5. Evaluation of Photocatalytic Activity

The photocatalytic activity of the resulting samples was investigated by oxidation of NO at ppb levels in a continuous flow reactor at ambient temperature. The volume of the rectangular reactor, which was made of stainless steel and covered with Saint-Glass, was 4.5 L (30 × 15 × 10 cm). A 150 W commercial tungsten halogen lamp was vertically placed outside the reactor above the reactor. Adequate distance was also kept from the lamp to the reactor for the same purpose to keep the temperature at a constant level. For the visible light photocatalytic activity test, UV cutoff filter (420 nm) was adopted to remove UV light. For each photocatalytic activity test experiment, two sample dishes (with a diameter of 12 cm) containing the photocatalyst powders was placed in the center of the reactor. The photocatalyst samples were prepared by coating an aqueous suspension of the samples onto the glass dish and then dried at 60 °C. The weight of the photocatalysts used for each dish was kept at 0.10 g. The NO gas was acquired from a compressed gas cylinder at a concentration of 100 ppm of NO (N_2_ balance). The initial concentration of NO was diluted to 550 ppb by the air stream supplied by purified air. The desired relative humidity (RH) level of the NO flow was controlled at 60% by passing the zero air streams through a humidification chamber. The gas streams were premixed completely by a gas blender, and the flow rate was controlled at 2.4 L/min by a mass flow controller. After the adsorption—desorption equilibrium was achieved, the lamp was turned on. The concentration of NOx was continuously measured by a chemiluminescence NO_x_ analyzer (42i-TL, Thermo Environmental Instruments Inc., Waltham, MA, USA), which monitors NO, NO_2_, and NOx (NOx represents NO + NO_2_) with a sampling rate of 1.0 L/min. The NO removal ratio (η) was calculated as η (%) = (1 − C/C_0_) × 100%, where C and C_0_ are concentrations of NO in the outlet steam and the feeding stream, respectively.

## 4. Conclusions

A simple ion-exchange method was developed for Br-doped BiOCOOH nanosheets. After Br-doping, the light absorption spectra of BiOCOOH were extended into the visible light region. The PL and photocurrent generation implied that Br-doping can improve separation of photogenerated electron/hole pairs. The Br-doped BiOCOOH exhibited enhanced visible-light photocatalytic removal of NO in air, which can be ascribed to the increased visible light absorption and the promoted charge separation. The concept of promoting visible-light photocatalysis through anion-exchange could also be extended to other layered semiconductor photocatalysts.
